# Novel Biocompatible Au Nanostars@PEG Nanoparticles for In Vivo CT Imaging and Renal Clearance Properties

**DOI:** 10.1186/s11671-017-2332-1

**Published:** 2017-10-12

**Authors:** Daiyuan Tang, Wei Gao, Yajiang Yuan, Lingling Guo, Xifan Mei

**Affiliations:** The First Affiliated Hospital of Jinzhou Medical University, Jinzhou, 121001 China

**Keywords:** AuNS@PEG nanoparticles, CT imaging, Renal clearance, Biocompatible

## Abstract

Nanoprobes are rapidly becoming potentially transformative tools on disease diagnostics for a wide range of in vivo computed tomography (CT) imaging. Compared with conventional molecular-scale contrast agents, nanoparticles (NPs) promise improved abilities for in vivo detection. In this study, novel polyethylene glycol (PEG)-functionalized Au nanoparticles with star shape (AuNS@PEG) with strong X-ray mass absorption coefficient were synthesized as CT imaging contrast agents. Experimental results revealed that AuNS@PEG nanoparticles are well constructed with ultrasmall sizes, effective metabolisability, high computed tomography value, and outstanding biocompatibility. In vivo imaging also showed that the obtained AuNS@PEG nanoparticles can be efficiently used in CT-enhanced imaging. Therefore, the synthesized contrast agent AuNS@PEG nanoparticles as a great potential candidate can be widely used for CT imaging.

## Background

The past decade has witnessed the rapid development of nanoparticles in nano-biotechnology, owing to their diverse constituent materials and large surface area [1, 2]. Among these nanoparticles, Au has a widely applications as its excellent biocompatibility and affinity in the biomedical field [3, 4]. In recent few years, Au nanoparticles are widely used in CT imaging, due to its bigger atomic number, precious metal, and chemical inertness, as well as not easy to proteins in the body reaction [5–7].

CT imaging is a noninvasive clinical diagnostic tool through different density and thickness of different tissues or organs of the X-ray generator attenuation in varying degrees, to form different tissue or organ distribution of gray-scale image contrast, and thus to the relative position of the lesion, and the size of the shape change [8–11]. Currently, the clinical application of CT contrast agents mainly contain iodine compound which is a small molecule including organic iodine and inorganic iodine small-molecule compounds, such as diaztrizoate (diatrizoic acid (DTA)) and iohexol (Omnipaque) [12]. However, the small molecular iodine-based contrast agent removing effects of iodine-containing compounds only needs a very short imaging time, and it does not have low kidney toxicity [13, 14]. In clinical practice, a deterioration of renal function is one complication of iodinated radiocontrast agents [15]. Therefore, the development of nano-materials provides new ideas and methods to solve these problems. Recent studies have also confirmed that nanoparticle-based CT contrast agent can effectively extend the imaging time, weakening of the kidney toxicity, and have better X-ray attenuation than iodine-based contrast agents, such as gold nanoparticles and nano-silver particles used as CT contrast agents have been attracted researchers attention [16–18]. Dendrimer nano-platform not only as modified small molecule of iodinated contrast media, but also as a template package and stability of different inorganic nanoparticles, improve blood circulation time of the contrast agent which make it better for CT imaging [19].

In this study, we prepared the PEG-functionalized Au nanostar nanoparticles (AuNS@PEG); due to the larger surface area in comparison with normal Au nanoparticles in the same size, Au nanostar could greatly enhance the CT imaging. After functionalized with PEG, Au nanostar nanoparticles could improve its biocompatible and renal clearance properties. Various methods, including TEM, EDX, XPS, MTT, and flow cytometry, were used to determine the characters and biocompatibility of AuNS@PEG nanoparticles. In addition, histological analysis and hematology studies had been used for tests about the toxicity of AuNS@PEG nanoparticles in vivo, and the results confirmed the nice biocompatible of AuNS@PEG nanoparticles. Moreover, in vitro and in vivo CT imaging experiments also exhibited the excellent CT imaging capabilities of AuNS@PEG nanoparticles. All of these results revealed that the synthesized contrast agent AuNS@PEG nanoparticles as a great potential candidate could be widely used for CT imaging and had good renal clearance properties.

## Methods

All experimental protocol including any relevant details were approved by the Regional Ethics Committee, Jinzhou Medical University, Liaoning Province, China.

### Materials and Instruments

All chemicals were purchased from Sigma-Aldrich (St. Louis, MO) and used directly unless otherwise noted.

Synthesized nanoparticles were characterized by transmission electron microscopy (TEM) and energy dispersive X-ray (EDX) analyses using 200-kV acceleration voltage (Tecnai G2 Twin, FEI, Hillsboro, OR). The TEM sample was prepared by drying diluted nanoparticle solutions on a formvar/carbon-coated copper grid. The samples were prepared by depositing a drop of a diluted colloidal solution on a carbon grid and allowing the liquid to dry in air at room temperature. UV-vis adsorption spectra were recorded on a Shimadzu UV-2450 UV/Vis/NIR spectrophotometer. Dynamic light scattering (DLS) measurement was performed on a Malvern Zetasizer NANO ZS at 25 °C.

### Synthesis of Au Nanostars/PEG (AuNS@PEG) Nanoparticles

Au nanostars (Au NS) were synthesized via seed-mediated growth method according to a previous report [20–22] with some slight adjustments. Typically, Au seeds which were formed with 10-nm diameter were synthesized by the chemical reduction of HAuCl_4_ according to previous report [23]; 6 ml HAuCl_4_ solution (*w*/*v* 1%) was added to 140 ml ultrapure water and heated to boiling while stirring. Then, 0.75 ml of oleylamine was rapidly injected, and the resulting mixture was boiled for another 2 h. The Au colloid was naturally cooled down to room temperature; 60 ml of cyclohexane was added to the colloid, and the solution was magnetically stirred for another 1 h. Subsequently, 1.5 ml of NaOH (4 M) was injected into the mixture while vigorously stirring for another 30 min. The mixture was left to be hierarchical. The Au nanoseed contained in the upper layer was precipitated by adding ethanol. The precipitates were alternately purified with ethanol and water one more time and dispersed in water.

Au nanostars with around 50-nm diameter were synthesized according to previous work by rapidly and simultaneously mixing AgNO_3_ (1 ml, 3 mM) and ascorbic acid (500 μl, 0.1 M) with 100 ml of a solution containing 0.25 mM HAuCl_4_, 1 mM HCl, and 1.5 ml of the gold nanosphere seeds. Then, the thiolated polyethylene glycol (PEG, 6 kDa) polymer was added in large excess to passivate the nanoparticle surface. The mixture solution was continuously stirred for 24 h, then the obtained AuNS@PEG nanoparticles were collected through 3 cycles of centrifugation/redispersion in water. The formed AuNS@PEG nanoparticles were redispersed in water for further use.

### Cell Culture and AuNS@PEG Nanoparticle Exposure

Neuroglia cells were collected from rat spinal cord tissues. The cells were cultured in Dulbecco’s modified Eagle’s medium (DMEM) (Gibco, USA) supplemented with 10% fetal bovine serum, 100 U per ml penicillin, and 100 μg per ml streptomycin at 37 °C in a humidified incubator with 5% CO_2_. Cells were seeded in culture plates followed by exposure to AuNS@PEG nanoparticles for 2 h at certain concentrations (50, 100, 200, 500, and 1000 ppm). DMEM without AuNS@PEG nanoparticles were used as the control group.

### Animals and Treatment

This work was carried out in strict accordance with the recommendations in the Guide for the Care and Use of Laboratory Animals of the National Institutes of Health. The protocol was approved by the Committee on the Ethics of Animal Experiments of the Jinzhou Medical University (permit number: LMU-2013-368), China. Male Sprague Dawley rats (180–200 g) were purchased from Animal Centre of the Jinzhou Medical University (license number: SCXK 2009-0004). All rats were fed in a temperature-controlled room (25.0 ± 0.2 °C) in a Specific Pathogen Free laboratory, with a 12-h/12-h light/dark photoperiod and 50% humidity. The rats were allowed free access to food and water.

Humane endpoints are chosen to minimize or terminate the pain or distress of the experimental animals via euthanasia, including inhalant agents, noninhalant pharmaceutical agents, and physical methods, rather than waiting for their deaths as the endpoint. In this work, rats were divided into two groups: (1) control: rats were anesthetized by intraperitoneal injection of chloral hydrate solution (10 wt%), and then, 800 μL of phosphate-buffered saline were injected via the tail vein. (2) Test: rats were anesthetized by intraperitoneal injection of chloral hydrate solution (10 wt%), and then, 800 μL of AuNS@PEG nanoparticle solution (200 μg/ml) were injected via the tail vein. For the H&E study, the rats were sacrificed by cervical dislocation without prior anesthesia, and their hearts, livers, kidneys, spleens, and intestines were immediately dissected, stored at − 80 °C, and snap-frozen in isopentane on dry ice until further processed.

### Cell Viability Assay

Logarithmic-phase neuroglia cells were seeded on a 96-well plate at 1 × 10^4^ cells per well in 100 μl cell suspension. Phosphate-buffered saline (PBS) was added to the surrounding wells. The plate was incubated at 37 °C and 5% CO_2_ for 24 h to allow the cells to adhere. The cells were then allocated to four groups: cells in the control group were incubated in DMEM containing 10% fetal bovine serum; in the AuNS@PEG nanoparticle group, 0, 25, 50, 100, 200, 500, or 1000 ppm AuNS@PEG nanoparticles were added to the culture medium; cells were observed 24 h later under an inverted phase contrast microscope (Leica, Heidelberger, Germany). Subsequently, 20 μl MTT (Sigma, St. Louis, MO, USA) was added to each well for 4 h. The medium was removed, and the cells were incubated with 150 μl of dimethyl sulfoxide for 10 min at 37 °C. Optical density (OD) values were measured at 490 nm with a microplate reader (Bio-Rad, Hercules, CA, USA).

### Flow Cytometry

Cells were incubated in 6-well plates for 24 h, then grouped and treated as described above. A single-cell suspension was made using trypsin and centrifuged at 300*g* for 3 min. Following removal of the supernatant, cells were washed twice with precooled PBS and centrifuged in 1 ml annexin V (Tianjin Sungene Biotech Co, Ltd., Tianjin, China) for 10 min. Cells were adjusted to 10^5^/ml. Cell suspension was centrifuged and washed three times with PBS. Samples (100 μl) were added to Eppendorf tubes with 5 μl annexin V-APC (Tianjin Sungene Biotech Co., Ltd.) and 7-AAD (Tianjin Sungene Biotech Co, Ltd.) and mixed. The volume was made up to 500 μl with PBS, and the tubes were incubated at room temperature for 15 min in the dark. Apoptosis was quantified by flow cytometry (BD FACSCanto II, BD Becton Dickinson, San Jose, CA, USA). Cell apoptosis rate was calculated as follows: number of apoptotic cells/total number of cells × 100%.

### CT Imaging

CT imaging was acquired using 128-row 64-slice spiral CT produced by General Electric Company (GE). Imaging parameters were as follows: slice thickness is 0.625; medium is nude mice; tube energy, kvp, is 120 μA and 100 mA; CTDIVOL is 6.53 mGy; and radius is 4.8 cm. All animals were scanned in the cranial to caudal direction from the low chest to the pelvis. CT data were analyzed by images and after-treatment.

### Histological Analysis

The organs were removed and fixed in 4% paraformaldehyde, then with 30% paraformaldehyde sucrose solution once every 2 days, sectioned, and stained with hematoxylin and eosin (H&E) for histological examination using standard techniques. The sections were examined under an inverted phase contrast microscope**.**

### Assessment of Renal Function

Biochemical analyzer (Jinzhou medical university) were used to evaluate BUN, Crea, β_2_-MG, and CO_2_ in the blood. Kidney function was evaluated by the changes of serum levels of BUN, Crea, β_2_-MG, and CO_2_ before and after injection of AuNS@PEG nanoparticles on rat.

### Statistical Analysis

Data were expressed as the mean ± SD and were analyzed using GraphPad Prism 5.0 software (GraphPad Software, Inc., La Jolla, CA, USA) and SPSS. Groups were compared using one-way analysis of variance and the least significant difference test. *P* < 0.05 was considered statistically significant.

## Results and Discussion

### Synthesis and Characterization of the AuNS@PEG Nanoparticles

Nanomaterials enter the human body and play the role of detection. The physical and chemical properties of the nanoparticles are first considered before they enter into the circulatory system [24, 25]. As we know, there are two key factors in the development of high-performance nanoprobes for in vivo CT imaging and renal clearance properties. One is further surface functionalization; the other is the size control.

**Fig. 1:**
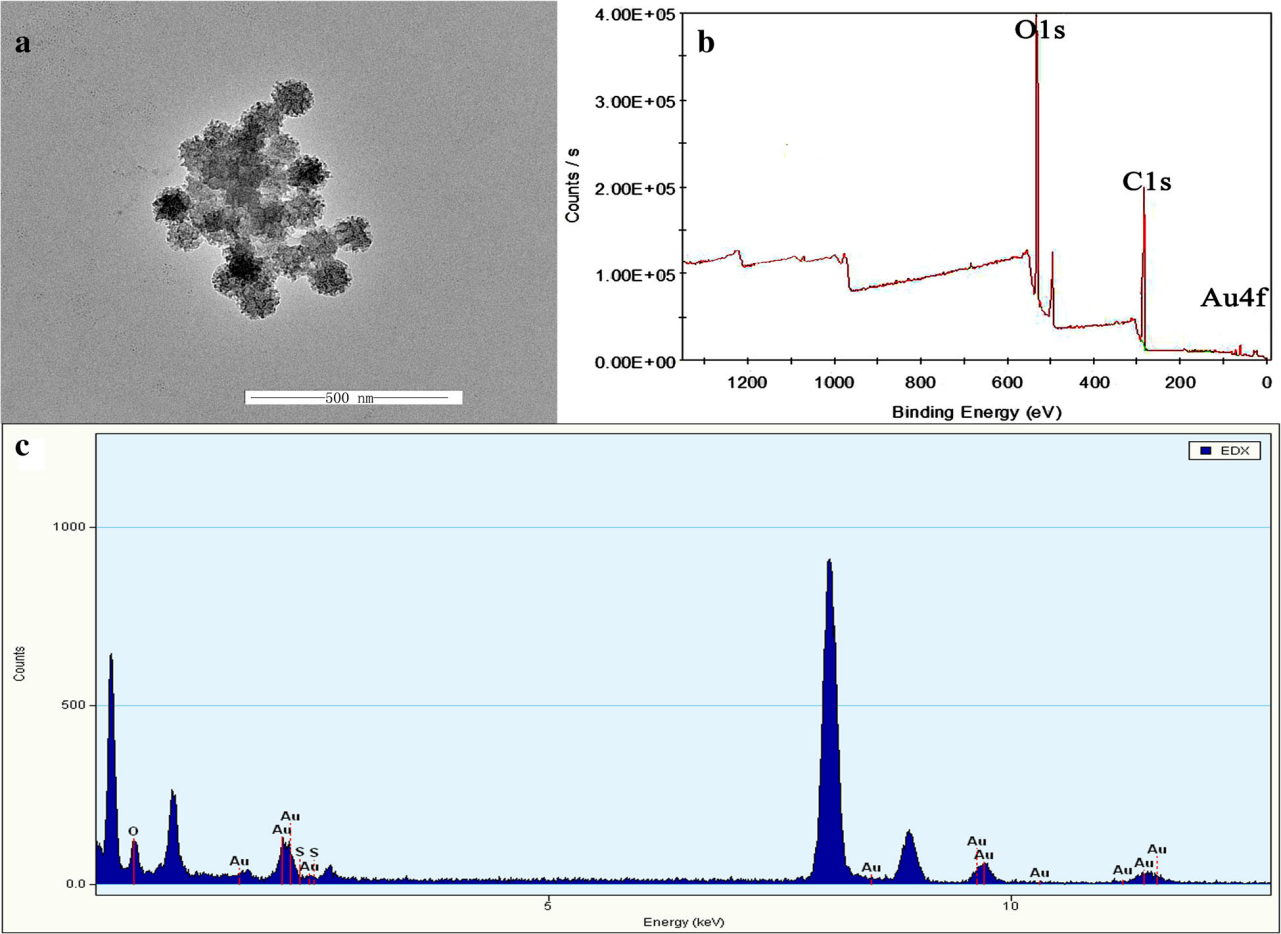
Transmission electron micrographs of AuNS@PEG nanoparticles (**a**), XPS (**b**), and EDX (**c**) of the AuNS@PEG nanoparticles

A large-scale transmission electron microscopy (TEM) image (Fig. 1a) was used to confirm the structure of AuNS@PEG nanoparticles, which showed obvious the star-structure AuNS@PEG nanoparticles were prepared, and these nanoparticles had the ideal sizes around 50 nm with high uniformity. Then, the elements of Au found in the energy dispersive X-ray (EDX) spectrum of AuNS@PEG nanoparticles also prove the preparation of Au nanostar (Fig. 1c). In addition, the composition on the surface of the AuNS@PEG nanoparticles was further characterized by XPS spectra, and the Au4f, C1s, and O1s derived from Au nanostars and PEG were clearly shown in the Fig. 1b which also confirm the formation of AuNS@PEG nanoparticles.

The above characteristics demonstrated the successful synthesis of AuNS@PEG nanoparticles.

### *CT* Value of the AuNS@PEG Nanoparticles

**Fig. 2:**
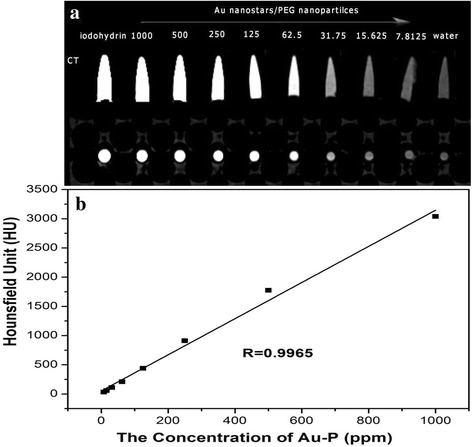
The X-ray attenuation intensity of the AuNS@PEG nanoparticles as a function of the Au concentration (**a**) and CT image of the AuNS@PEG nanoparticles under different concentrations (iodohydrin, 1000, 500, 250, 125, 62.5, 31.75, 15.625, and 7.8125 ppm, respectively) (**b**)

Au nanoparticles have been widely used as CT contrast agents because of their better X-ray attenuation property than conventional iodine-based small-molecule CT contrast agents. Iodine (*Z* = 53) has historically been the atom of the first choice in CT imaging field. To assess the feasibility of AuNS@PEG nanoparticles for X-ray computed tomography imaging, we measured the CT values (Hounsfield units, HU). Figure 2a shows that the AuNS@PEG nanoparticles have higher CT value compared to the iodine and DI water at the same concentration. When the AuNS@PEG nanoparticle concentration increased, the CT image intensity also continuously increased with brighter images. By plotting the CT value (in HU) of the AuNS@PEG as function of concentration (Fig. 2b), we could see a linear attenuate of the CT value of AuNS@PEG nanoparticles with the different concentrations. These results reveal that AuNS@PEG nanoparticles are ideal candidates for a positive CT imaging nanoprobe.

### Cytotoxicity Assay

**Fig. 3:**
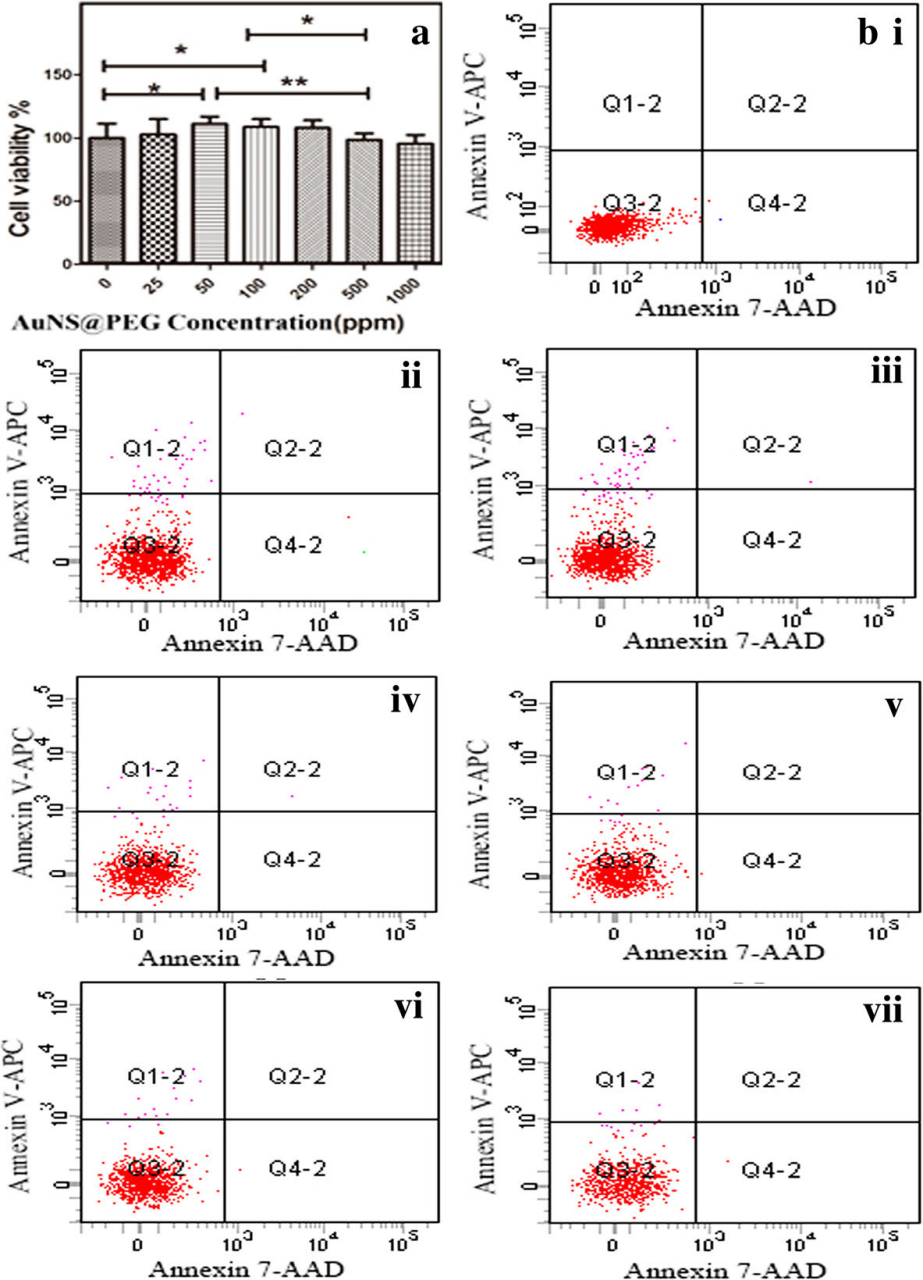
Cell viability of neuroglia incubated with different concentrations of AuNS@PEG nanoparticles for 24 h (**a**); the apoptosis of cells induced by AuNS@PEG nanoparticles is shown by flow cytometry: control (**bi**), 25 ppm (**ii**), 50 ppm (**iii**), 100 ppm (**iv**), 200 ppm (**v**), 500 ppm (**vi**), and 1000 ppm (**vii**)

It was crucial to investigate the biocompatibility of AuNS@PEG nanoparticles in vitro before it was used in CT imaging in vivo as a contrast agent. MTT assay was performed to evaluate their cytotoxicity on neuroglia cells. After incubation with AuNS@PEG nanoparticles at different concentrations (25, 50, 100, 200, 500, and 1000 ppm, respectively) for 24 h, an MTT viability assay of neuroglia cells was carried out. It could be seen that the viability of the cells after treatment with AuNS@PEG nanoparticles in the studied concentration range is quite similar to the control, which clearly indicated that the formed AuNS@PEG nanoparticles have a good cytocompatibility at a concentration up to 200 ppm. Even at a relatively high dose of nanoparticles (1000 ppm), the cell viability still remained above 90% (Fig. 3a).

The cytocompatibility of the AuNS@PEG nanoparticles was further confirmed by flow cytometric analysis of the cells treated with the AuNS@PEG nanoparticles at different concentrations for 2 h. In the flow cytometric analysis, cells were stained with annexin V-APC and 7-AAD after treatment with PBS and AuNS@PEG nanoparticles. Neuroglia cells treated with PBS without staining was used as the control (Fig. 3bi). It could be seen that cells treated with the AuNS@PEG nanoparticles at concentrations of 25, 50, 100, 200, 500, and 1000 ppm, respectively (Fig. 3bi–vii). Taken together with the results from MTT assay, our results exhibited that the AuNS@PEG nanoparticles have good cytocompatible, and there was no obvious cellular morphology change after treatment with the AuNS@PEG nanoparticles, which agreed with the MTT data.

### In Vivo CT Imaging and Biodistribution

**Fig. 4:**
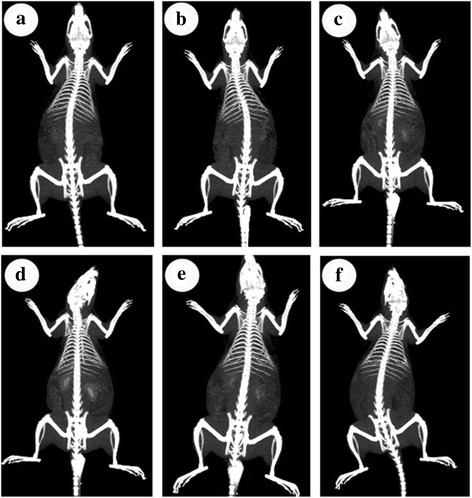
CT images of rat before injection (**a**) and at different time points (0.5, 1, 2, 6, and 24 h) (**b**–**f**) after intravenous injection of AuNS@PEG nanoparticles (200 ppm)

Encouraged by their high CT contrast performance in the in vitro experiment, we have further confirmed the feasibility of AuNS@PEG nanoparticles as a CT contrast agent in vivo. AuNS@PEG nanoparticles (200 ppm) were injected intravenously into the tail veins of the rat. Such a dose of the AuNS@PEG nanoparticles was chosen because of the results of low toxicity and apoptosis percentage of MTT and flow cytometry and high sensitivity of CT. The CT imaging of the important organ regions were recorded before tail vein injection and at different time points post tail vein injection (Fig. 4). Our study aim to test the capacity of CT imaging and renal clearance. So we stress the change of the organ of kidney and bladder in the CT imaging. Figure 4a is the CT image of the rat kidney before injection. Compared with pre-injection, the kidney imaging is greatly enhanced from 0.5 to 2 h (Fig. 4b–d). The time-dependent distribution of the AuNS@PEG nanoparticles in the rat was also tracked by CT signal value after intravenous injection. The kidney and bladder imaging were greatly enhanced from 0.5 to 2 h, and HU value of them rose from 95 to 464 and 105 to 664. After 6 h post-injection, the CT contrast intensity in the kidney of rat obviously decrease over time (Fig. 4e). After 24 h post-injection, CT imaging of bladder organ is completely clear, showing the excellent renal clearance properties of AuNS@PEG nanoparticles (Fig. 4f). Owing to their optimal particle size and surface functionalization, the elimination of AuNS@PEG nanoparticles from blood during circulation can be such slow. Hence, these results indicate that the as-prepared AuNS@PEG nanoparticles might be a unique and promising nanoprobe to provide the real-time CT imaging in vivo. This is beneficial for future clinical applications because the contrast agents can be administered to patients in the hospital.

### H&E Staining

**Fig. 5:**
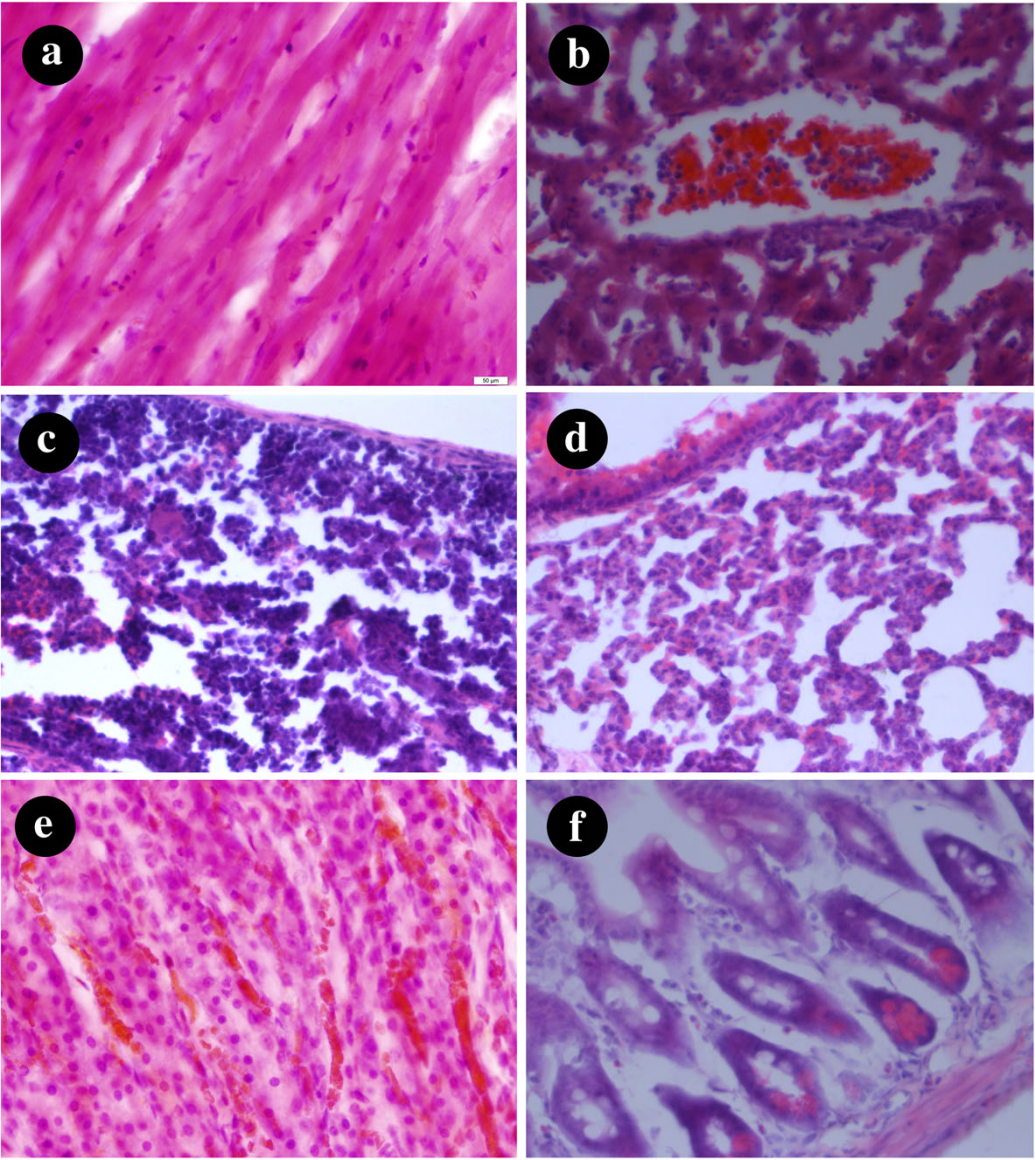
Tissue sections stained with H&E: **a** heart, **b** liver, **c** spleen, **d** lung, **e** kidney, and **f** intestine. The scale bars represent 100 mm

Histological changes in the organs of the mice were performed after 24 h post-injection of AuNS@PEG nanoparticles, and the results were shown in the Fig. 5, We can see that no obviously change in the histology of the major organs was observed, and most important, there are no residual AuNS@PEG nanoparticles left in these organs. Based on the above results, the AuNS@PEG nanoparticles exhibited good biocompatibility and no obvious in vivo toxicity, which promise it as a new CT imaging contrast agent for biological medicine application.

### Renal Function Study of AuNS@PEG Nanoparticles

To further evaluate the in vivo toxicity of AuNS@PEG nanoparticles, the parameters of BUN, Crea, β_2_-MG, and CO_2_ were measured for studies of renal function; we analyzed the serum. These values can assess the renal function of the rat is good or not. The values of BUN can assess the rat’s urinary function. The changing value of Crea represents the various diseases in the rat’s body. The β_2_-MG concentration is mainly related to renal tubular function. And the value of CO_2_ can evaluate the acidification function of the renal tubule. Rat was given AuNS@PEG nanoparticles at a concentration of 200 ppm. The level of these results was examined 24 h after injection, and there were no difference between before and after injection of AuNS@PEG nanoparticles in rat (Table 1).

**Table 1:** The effect of AuNS@PEG nanoparticles on BUN, Crea, β_2_-MG, and CO_2_ levels before and after injection of AuNS@PEG nanoparticles in rat (*n* = 5)

	Bun (mmol/l)	Crea (μmol/l)	β_2_-MG (mg/l)	CO_2_ (mmol/l)
Before injection	10.17 ± 0.09	28.00 ± 1.00	0.30 ± 0.10	22.16 ± 1.49
After injection	9.12 ± 0.87	26.33 ± 2.51	0.50 ± 0.10	17.80 ± 1.10

## Conclusions

In summary, we developed facile AuNS@PEG nanoparticles for applications in CT imaging. The formed AuNS@PEG nanoparticles have ultrasmall sizes, low toxicity, good water dispersibility, hemocompatibility, and cytocompatibility in the given concentration range. The CT values show that the AuNS@PEG nanoparticles have a good bright imaging. In vitro imaging results indicate that the AuNS@PEG nanoparticles possess strong X-ray attenuation properties as a new contrast agent for CT imaging applications, which were also demonstrated by the CT imaging of rat kidney in vivo. Moreover, the distribution of biological study and exploration of in vivo toxicity show that AuNS@PEG nanoparticles can metabolize and have high biological compatibility. Thus, AuNS@PEG nanoparticles can be promising candidates for medical applications.
